# Case Report: Rheumatoid meningitis with positive NMDAR antibody: A case treated with intravenous immunoglobulin

**DOI:** 10.3389/fimmu.2022.971934

**Published:** 2022-10-07

**Authors:** Honglian Zhang, Yuehong Wan, Zhujun Mei, Chen Xie, Shiying Liu, Hongbing Nie, Fan Hu, Renshi Xu

**Affiliations:** Jiangxi Provincial People’s Hospital, The First Affiliated Hospital of Nanchang Medical College, Nanchang, China

**Keywords:** rheumatoid meningitis, IVIg, case report, diagnosis and treatment, NMDAR antibody

## Abstract

As a rare complication of rheumatoid arthritis (RA) in the central nervous system (CNS), rheumatoid meningitis (RM) mainly affects the meninges and has various clinical symptoms. The diagnostic and treatment approaches currently used are not practical. RM cases with positive NMDAR antibodies (Abs) have never been reported. In the present study, a 66-year-old man with a 1-year history of RA presented recurrent left lower limb weakness during activities for 1 month. The results showed that rheumatoid factor (RF) and anti-cyclic citrullinated peptide antibody (ACPA) were positive in the serum, and NMDAR Abs were present in cerebrospinal fluid (CSF). Hyperintensity was observed in the leptomeninges of the right frontal and parietal lobes, and subtle hyperintensity was observed in the left frontal and parietal lobes, as indicated by brain MRI. A meningeal biopsy revealed non-specific inflammation with the absence of rheumatoid nodules. The patient was given IVIg on day 7 after admission. The clinical symptoms were relieved, the lesions were alleviated, and abnormal biochemical indicators were gradually recovered 1 week after initiation of the treatment, while NMDAR Abs were present in CSF even after treatment. After 5 months of follow-up, the patient’s serum and CSF ACPA and IL-6 levels were still high. The findings showed that brain MRI was adequate for the diagnosis of RM. ACPA and IL-6 might be the specific biomarkers for disease activity in RM. IVIg was effective as induction therapy for RM. Further studies should explore whether the presence of NMDAR Abs is associated with RM.

## Introduction

As a rare complication of rheumatoid arthritis (RA), rheumatoid meningitis (RM) affects the central nervous system (CNS) (1, 2), such as the dura mater, pia mater, and arachnoid mater, with the main effects observed in the pia mater (1, 3–5). The first case of RM was reported approximately 70 years ago (6). However, a practical diagnostic approach for RM has not been elucidated. Furthermore, the clinical manifestations of RM markedly vary with no specific changes in biochemical indicators, imaging, and pathology, leading to the limited effectiveness of diagnosis (1, 4, 5, 7, 8). In addition, there is no well-laid guideline for RM treatment, and high-dose corticosteroid remains the conventional induction therapy for RM (1, 4). Notably, studies have not explored intravenous immunoglobulin (IVIg) as induction therapy for RM treatment.

Anti-N-methyl-D-aspartate receptor (anti-NMDAR) encephalitis is the most common form of autoimmune encephalitis caused by autoantibodies to the NMDA receptor (9). Tumors, pathogen infections, systemic autoimmune diseases (ADs), and other unknown factors trigger the release of Abs against NMDAR (9–11). Some systemic ADs, such as Hashimoto’s thyroiditis, systemic lupus erythematosus, urticaria, and allergic purpura, are associated with anti-NMDAR positivity (12, 13). However, the relationship between RM and positive NMDAR Abs has not been reported. In the current study, we presented a case of RM with positive NMDAR Abs. The patient was treated with IVIg as induction therapy.

## Case description

**Figure 1:**
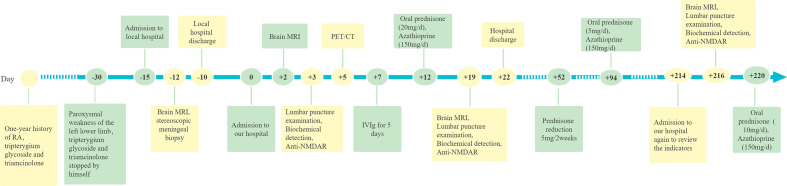
Timeline of the clinical manifestations and treatment progression.

**Figure 2:**
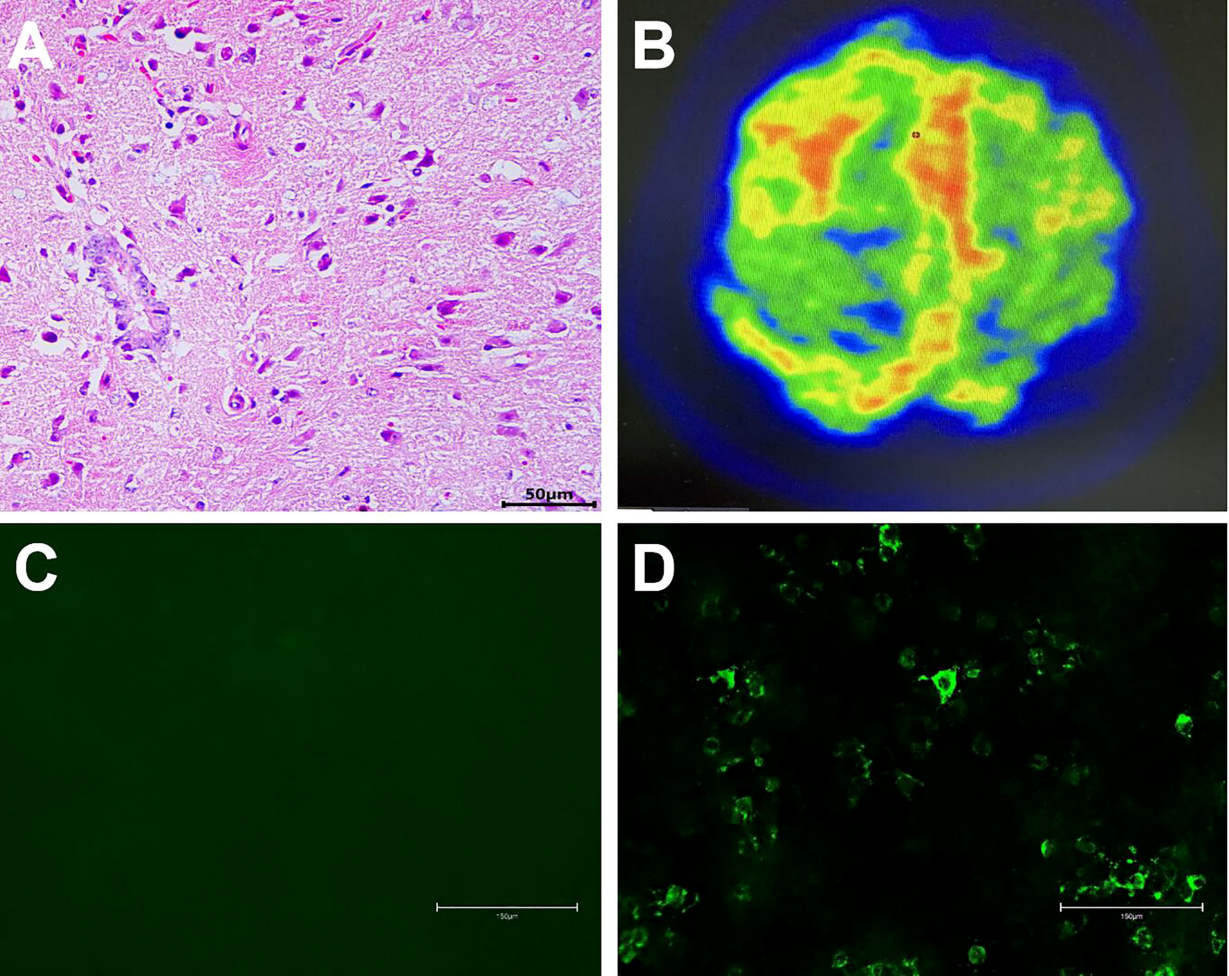
A non-specific inflammation and no rheumatoid nodules in the meninges (H&E, scale bar=50 µm, **(A)**. ^18^F-FDG PET/CT scan showed hypermetabolism of bilateral frontal-parietal meninges and adjacent cortex, especially on the right side **(B)**. NMDAR Abs were absent in serum (scale bar=150 µm, **(C)**), and NMDAR Abs (1:1) were detected in the CSF (scale bar=150 µm, **(D)**).

A 66-year-old man, who presented with paroxysmal weakness of the left lower limb during activities for half a month, was admitted to a local hospital in December 2021 (**Figure 1**). The patient reported one to two transient episodes per day with a duration of 2-10 min for each transient episode. The patient had a history of well-managed hypertension and a 1-year history of RA. This patient presented with pain in the major joints of the extremities without minor arthritis of the extremities and extra-articular manifestations, and he was treated with tripterygium glycoside tablets (three times per day, 25 mg a time) and triamcinolone tablets (8 mg/day). He stopped taking these medicines half a month before admission to the hospital because it was not responding to his joint pain. Magnetic resonance imaging (MRI) examination showed atypical enhancement of the pia meningeal, whereas stereoscopic meningeal biopsy only indicated non-specific inflammatory changes (**Figures 1**, **2A**). The patient was transferred to our hospital in January 2022 owing to a poor diagnosis (**Figure 1**).

**Figure 3:**
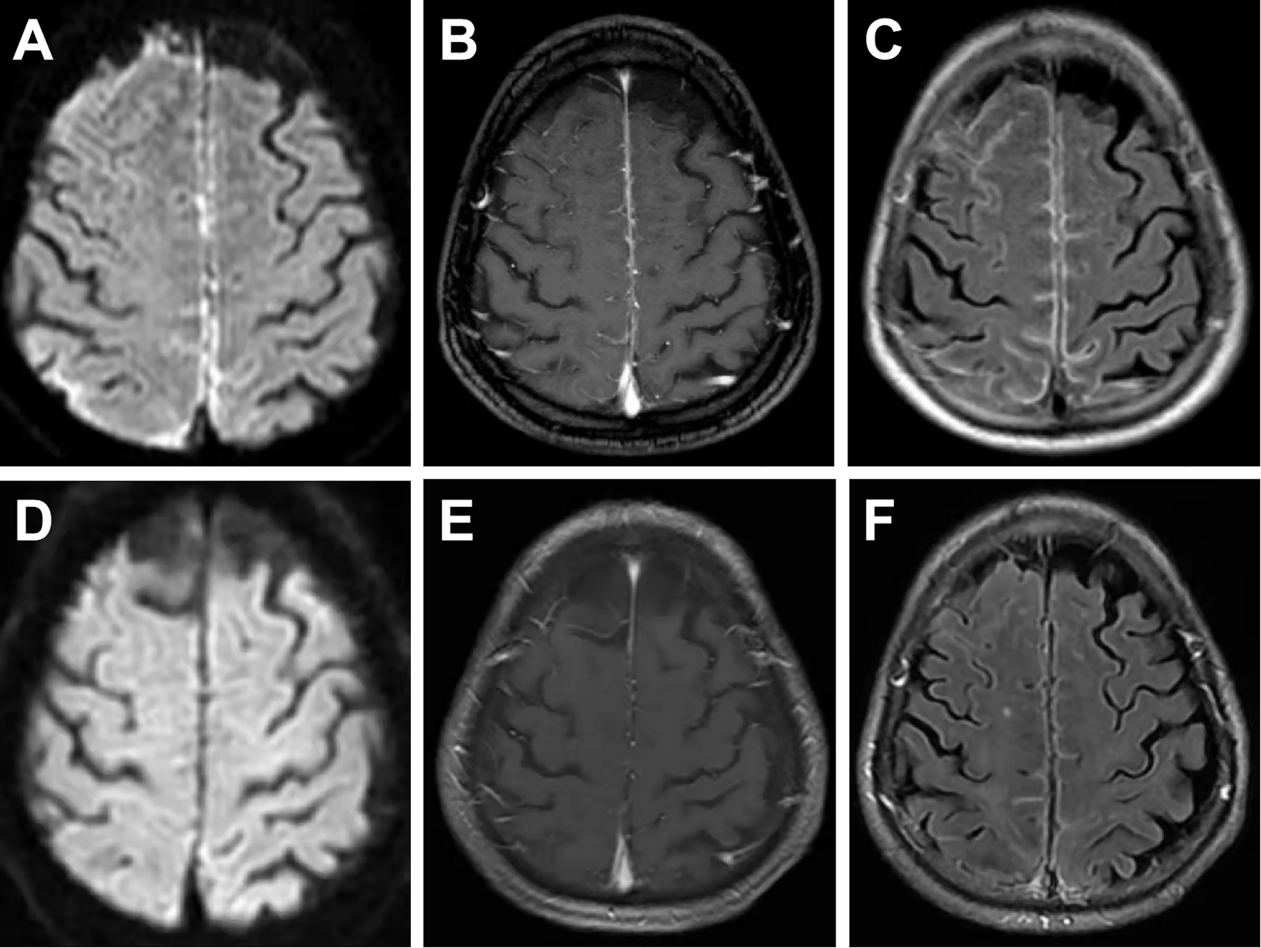
On the first admission, brain MRI showed evident hyperintensity in the leptomeninges of the right frontal and parietal lobes and subtle hyperintensity in the left frontal and parietal lobes in DWI images **(A)**, contrast-enhanced T1-weighted sequences **(B)**, and contrast-enhanced FLAIR sequences **(C)**. MRI conducted at a 1-month follow-up showed subtle hyperintensity of the bilateral frontal and parietal lobes in DWI images **(D)**, contrast-enhanced T1-weighted sequences **(E)**, and contrast-enhanced FLAIR sequences **(F)**.

On examination, the patient was in good general condition without headache, psychiatric behavior, cognitive dysfunction, speech dysfunction, seizures, movement disorder, consciousness disorder, and autonomic dysfunction. Neurological examination was rigorously normal except for a positive Babinski sign on the left lower limb. Musculoskeletal examination revealed only slight tenderness of large joints of extremities. Hyperintensity was observed in the leptomeninges of the right frontal and parietal lobes, and subtle hyperintensity was observed in the left frontal and parietal lobes as indicated in the diffusion-weighted image (DWI) (**Figure 3A**), contrast-enhanced T1-weighted sequences (**Figure 3B**), and contrast-enhanced fluid-attenuated inversion recovery (FLAIR) sequences (**Figure 3C**) after examination through brain MRI. No irregularity was observed in intracranial arteries through magnetic resonance angiography (MRA). The patient underwent ^18^F-FDG PET/CT scan, showing hypermetabolism of bilateral frontal-parietal meninges and adjacent cortex (**Figure 2B**). Electroencephalography (EEG) did not show any changes in brain activity. Cardiac color Doppler ultrasound, ECG, and CT examinations of the whole abdomen and lung were normal.

A lumbar puncture examination was performed on the 3^rd^ day after admission (**Figure 1**). Cerebrospinal fluid (CSF) analysis showed increased protein content (0.477 g/L), high pressure (260 mm H_2_O), and elevated cell count (60 cells/mm^3^, with high levels of lymphocytes, **Table 1**). Notably, concentrations of lactic acid, glucose, and chloride were normal, whereas viral and fungal markers and culture results using CSF were negative. Analysis showed positive RF (96.3 IU/mL) and ACPA (>800 U/mL). In addition, C-reactive protein (CRP, 13.30 mg/L) was increased, while the erythrocyte sedimentation rate (ESR, 14 mm/h) was normal. Flow cytometry showed that the concentration of serum IL-6 (37.51 pg/mL) was significantly increased (**Table 1**). Cell-based assay (CBA) showed the presence of NMDAR Abs IgG in CSF (titer 1:1, **Figure 2D**), while the Abs were absent in serum (**Figure 2C**). Moreover, other autoimmune Abs (ANA, ENA, ANCA, anti-thyroid, anti-AMPAR1, anti-AMPAR2, anti-LG1, anti-CASPR2, anti-GABABR, anti-DPPX, anti-IgLON5, anti-GABAAR α1, anti-GABAAR β3, anti-GlyRα1, anti-GluR5, anti-D2R, anti-Neurexin3α, and anti-GAD65) were not observed. Serum concentrations of C3, C4, IgA, IgG, and IgM were normal. Analysis showed negative results for infectious pathogens, such as herpesviruses, Mycobacterium tuberculosis, HIV, syphilis, and Lyme disease (**Figure 1**).

**Table 1:** Serum and CSF markers before and after treatment.

Test/(range)	Pre-treatment Day 3	Post-treatment Day 19	Post-treatment Day 216
Serum			
ESR (<15 mm/h)	14	−	8
CRP (<8.0 mg/L)	13.3↑	1.0	4.24
RF (<20 IU/mL)	96.3↑	57.5↑	<20
ACPA (<5 U/mL)	>800↑	>800↑	>800↑
IL-6 (<5.4 pg/mL)	37.51↑	9.62↑	14.79↑
NMDAR Ab	Negative	−	−
CSF			
Cell count (<5 E6/L)	60↑	20↑	10↑
Pressure (80~180 mmH_2_O)	260↑	185↑	155
Protein (0.15~0.45 g/L)	0.477↑	0.185	0.166
RF (<20 IU/mL)	−	−	<20
ACPA (<0.4 U/mL)	−	−	2.8↑
IL-6 (<5.4 pg/mL)	−	−	6.16↑
NMDAR Ab	(1:1)↑	(1:1)↑	(1:1)↑

The patient underwent a composite evaluation, and a diagnosis of RM with positive NMDAR Abs was proposed. He received IVIg (0.4 g/kg.d) for 5 days from the 7^th^ day after admission. Notably, the left lower limb weakness was alleviated after the second IVIg treatment. The patient received oral prednisone acetate (20 mg/day) and azathioprine (150 mg/day) to prevent the recurrence of RM (**Figure 1**). Analysis of the lumbar puncture, some biochemical indexes, and MRI examination were conducted after 7 days of IVIg treatment. CSF analysis showed that the protein content (0.185 g/L) was restored to the normal level, and the pressure (185 mmH_2_O) and the cell count (20 cells/mm^3^) were decreased compared with pre-treatment values. CRP level (1.0 mg/L) was normal, and IL-6 level (9.62 pg/mL) was significantly lower than that before treatment. Serum RF (57.5 IU/mL) and ACPA (>800 U/mL) were positive (**Table 1**). The titer of NMDAR Abs in CSF was 1:1, similar to that observed before treatment (**Table 1**). Only subtle hyperintensity of the bilateral frontal and parietal lobes was observed in DWI (**Figure 3D**), contrast-enhanced T1-weighted sequences (**Figure 3E**), and contrast-enhanced FLAIR sequences (**Figure 3F**). The patient was generally in good condition, and RM did not relapse during the 1-month follow-up. The patient was instructed to reduce the prednisone dose to 5 mg per day by 5 mg every 2 weeks (**Figure 1**). After 5 months, the patient was hospitalized for follow-up and was in good physical condition (**Figure 1**). Indicators of abnormal levels included IL-6 in serum (14.79 pg/mL) and CSF (6.16 pg/mL), ACPA in serum (>800 U/mL) and CSF (2.8 U/mL), and NMDAR Abs in CSF (1:1), while other indicators were basically normal (**Table 1**). Considering that the IL-6 and ACPA levels of the patient were still abnormal, he was suggested to increase the dosage of prednisone acetate tablet to 10 mg per day (**Figure 1**).

## Discussion

RA is the most common disease of connective tissues, which mainly affects the joints and sometimes extra-articular systems, such as the muscular system, CNS, peripheral nervous system, and so on (14). RM is an extremely rare neurological manifestation of RA, first described in 1954 by Ellman et al. (6). The clinical symptoms of RM are varied and non-specific, and the most frequently reported clinical symptoms include headache, seizures, encephalopathy, and/or neurologic deficits (1, 4, 5, 7). RM should be considered when the patient has a history of RA, RF or ACPA is positive, and other pathogenic causes are excluded (1, 4). Nonetheless, RM still remains difficult to diagnose. Theoretically, a pathological biopsy of the meninges is required for a definite diagnosis (1). However, the meninges in most RM cases are featured by non-specific inflammation or granulomatous necrosis through biopsy or autopsy (15, 16). Brain MRI with asymmetrical meningeal involvement has been reported to be helpful for the diagnosis of RM (1, 4, 8).

As a transmembrane ion channel type glutamate receptor, NMDAR is mainly expressed in nerve cells of the brain and is implicated in modulating brain plasticity, learning, and cognitive function (9). NMDAR has three different subunits, namely NR1, NR2, and NR3 (17). Anti-NMDAR IgG mainly targets the NR1 subunit of NMDAR in the postsynaptic membrane of neurons. Notably, anti-NMDAR IgG is most commonly detected in anti-NMDAR encephalitis (18). The main clinical manifestations of anti-NMDAR encephalitis include psychosis, cognitive decline, epileptic seizures, dyskinesia, decreased consciousness, and autonomic instability (9, 19). As for the diagnosis of anti-NMDAR encephalitis, there are reliable criteria that assist in identifying the disease on clinical grounds and highly specific antibody tests to confirm the clinical diagnosis (9, 19). Although the patient we reported had positive anti-NMDAR IgG in CSF, he lacked typical clinical manifestations and abnormal EEG, which could not be diagnosed as anti-NMDAR encephalitis according to Graus and Dalmau criteria (19).

In the RM patient we reported, CSF NMDAR Abs were only weakly positive, and he showed no symptoms of AE. Therefore, the relation between RM and NMDAR Abs remained uncertain. One possibility was that such positivity was a false positive. However, a subclinical synthesis of NMDAR Abs could not be excluded. The compromised blood-brain barrier (BBB) of RM might play a vital role in the pathological process, allowing substantial access of NMDAR Ab-specific plasma cells to the brain and then producing specific Abs under the action of various cytokines, such as C-X-C motif chemokine 13 (CXCL-13) and IL-6 (9, 20, 21). CXCL-13 is a B lymphocyte chemokine, and IL-6 is a cytokine produced by activated T cells and fibroblasts, enabling B cell precursors to become Ab-producing cells (9). Elevated CXCL-13 and IL-6 levels in CSF are associated with NMDAR Ab generation through interacting with B lymphocytes (20, 21). We and other reports found elevated levels of CXCL-13 and IL-6 in CSF of RM patients, which might also be responsible for NMDAR Ab positivity in CSF of RM (4, 22, 23). However, the long-term use of immunosuppressants in the patient we reported might be responsible for the only subclinical synthesis of NMDAR Abs and the absence of clinical symptoms of AE. We will continue to follow the changes of CSF NMDAR Abs and the progress of the patient’s disease, which might progress to AE in the future.

^18^F-FDG PET/CT scan is an emerging imaging adjunct mostly applied in the study of neurodegenerative disorders and demonstrated to be also useful in the evaluation of brain inflammatory diseases, such as AE and meningitis (9). Reports of ^18^F-FDG PET/CT on anti-NMDAR encephalitis are mainly small case series that demonstrate various degrees of hyper- or hypometabolism of a particular area of the brain, which is associated with different stages of anti-NMDAR encephalitis and the different structures involved at each stage (9, 24, 25). The metabolism of the frontotemporal lobe is inconsistent in different reports, some are hypermetabolism, and some are hypometabolism. However, medial occipital hypometabolism and striatal hypermetabolism are consistent in various reports (24–26). Hypermetabolism of meninges and adjacent cortex is a typical feature of meningitis shown by ^18^F-FDG PET/CT (27). In the current study, ^18^F-FDG PET/CT scan showed that the hypermetabolism of bilateral frontal-parietal meninges and adjacent cortex, especially on the right side, was more likely the supportive evidence of meningitis rather than anti-NMDAR encephalitis.

ACPA is a specific indicator for the diagnosis of RA with a specificity of 96% (28), while there is no specific biomarker for RM until now. It has been reported that the levels of ACPA in serum and CSF of RM are elevated and then significantly decreased after immunosuppressive therapy (23, 29–31). In our case, ACPA levels in serum and CSF were also increased. Therefore, ACPA might be a useful marker to diagnose RM as well as to assess the efficacy of treatment, which needs to be further confirmed by more reports. High levels of IL-6 in CSF suggest an ongoing intense inflammatory process in the CSF/meninges (31). Previous findings indicate that IL-6 levels in CSF are associated with the activity and pathogenesis of RM (30, 31). Therefore, the potential use of IL-6 in CSF may also serve as a specific biomarker for disease activity in RM. Since the patient’s serum and CSF ACPA and IL-6 levels were still high during the last follow-up, suggesting that RM might still be active, we increased the dose of his prednisone acetate from 5 mg to 10 mg.

Current approaches for RM treatment have various limitations. Immunosuppressive therapy is currently the main treatment regimen for ADs, mainly used to alleviate systemic inflammation and prevent permanent damage caused by these diseases (12). RM treatment, like other ADs, consists of induction and maintenance therapy (1). High-dose intravenous methylprednisolone (1 g/day for 3-5 days) as induction treatment presents good outcomes in most RM patients. Studies report that other immunosuppressive drugs, such as ciclosporin, methotrexate, azathioprine, mycophenolate mofetil, and cyclophosphamide, can be used for the treatment of RM patients (8, 32–35). IVIg is a therapeutic preparation of IgG obtained from the plasma of healthy people. IVIg was initially used for the treatment of primary and secondary immunodeficiency and is currently used for the management of ADs (36). Only very few reports are available on the efficacy of IVIg in the treatment of RM. In the current case, IVIg rapidly relieved the clinical symptoms of the patient, the imaging lesions were alleviated, and abnormal biochemical indicators were gradually recovered 1 week after the treatment was initiated, indicating that IVIg was effective as the induction treatment of RM. Notably, low doses of prednisone acetate combined with other immunosuppressive drugs should be used as maintenance treatment of RM to effectively prevent the recurrence of the disease (1, 4, 8).

In summary, RM was a rare complication of RA in CNS, mainly affecting the meninges and having various clinical symptoms. Further studies are still required to clarify whether the positivity of NMDAR Abs in RM was a mere coincidence or if there was a pathogenic link. ACPA and IL-6 might be the specific biomarkers for disease activity in RM. IVIg was effective as induction therapy for RM, and low-dose prednisone acetate combined with other immunosuppressive drugs was required as maintenance therapy. The shortcomings of this paper were that the concentrations of IL-6, ACPA and RF in CSF were only detected after treatment and follow-up time of patient was not long enough.

## Patient perspective

At first, I didn’t pay attention to the onset of paroxysmal weakness in my left lower limb, but similar symptoms recurred. I gradually became afraid and attended the hospital to seek treatment. I am very grateful to my doctors for effectively diagnosing the disease. The paroxysmal weakness in my left lower limb was alleviated after IVIg treatment. My disease has the risk of developing into AE. Therefore, I need to make regular follow-up visits, take medicine regularly, pay attention to rest, and avoid fatigue in the future to reduce this risk.

## Data availability statement

The original contributions presented in the study are included in the article/Supplementary Material. Further inquiries can be directed to the corresponding author.

## Ethics statement 

The studies involving human participants were reviewed and approved by Medical ethics committee of Jiangxi Provincial People’s Hospital. The patients/participants provided their written informed consent to participate in this study. Written informed consent was obtained from the individual(s) for the publication of any potentially identifiable images or data included in this article.

## Author contributions

HZ and RX drafted and revised the manuscript. YW, ZM, CX, SL, HN, and FH collected data and interpreted the data. All authors contributed to the article and approved the submitted version.

## Funding

The publishing of this work was financed by Jiangxi Provincial Health and Family Planning Commission.

## Acknowledgments

The authors thank the patient for participation in the study.

## Conflict of interest

The authors declare that the research was conducted in the absence of any commercial or financial relationships that could be construed as a potential conflict of interest.

## Publisher’s note

All claims expressed in this article are solely those of the authors and do not necessarily represent those of their affiliated organizations, or those of the publisher, the editors and the reviewers. Any product that may be evaluated in this article, or claim that may be made by its manufacturer, is not guaranteed or endorsed by the publisher.
